# Diversity of wetland plants used traditionally in China: a literature review

**DOI:** 10.1186/1746-4269-10-72

**Published:** 2014-10-15

**Authors:** Yin Zhang, Hualin Xu, Hui Chen, Fei Wang, Huyin Huai

**Affiliations:** College of Bioscience and Biotechnology, Yangzhou University, Yangzhou, 225009 China

**Keywords:** Wetland plants, Traditional knowledge, Literature study, China

## Abstract

**Background:**

In comparison with terrestrial plants, those growing in wetlands have been rarely studied ethnobotanically, including in China, yet people living in or near wetlands can accumulate much knowledge of the uses of local wetland plants. A characteristic of wetlands, cutting across climatic zones, is that many species are widely distributed, providing opportunities for studying general patterns of knowledge of the uses of plants across extensive areas, in the present case China. There is urgency in undertaking such studies, given the rapid rates of loss of traditional knowledge of wetland plants as is now occurring.

**Methods:**

There have been very few studies specifically on the traditional knowledge of wetland plants in China. However, much information on such knowledge does exist, but dispersed through a wide body of literature that is not specifically ethnobotanical, such as regional Floras. We have undertaken an extensive study of such literature to determine which species of wetland plants have been used traditionally and the main factors influencing patterns shown by such knowledge. Quantitative techniques have been used to evaluate the relative usefulness of different types of wetland plants and regression analyses to determine the extent to which different quantitative indices give similar results.

**Results:**

350 wetland plant species, belonging to 66 families and 187 genera, were found to have been used traditionally in China for a wide range of purposes. The top ten families used, in terms of numbers of species, were Poaceae, Polygonaceae, Cyperaceae, Lamiaceae, Asteraceae, Ranunculaceae, Hydrocharitaceae, Potamogetonaceae, Fabaceae, and Brassicaceae, in total accounting for 58.6% of all species used. These families often dominate wetland vegetation in China. The three most widely used genera were *Polygonum*, *Potamogeton* and *Cyperus*. The main uses of wetlands plants, in terms of numbers of species, were for medicine, food, and forage. Three different ways of assigning an importance value to species (Relative Frequency of Citation RFC; Cultural Importance CI; Cultural Value Index CV) all gave similar results.

**Conclusions:**

A diverse range of wetland plants, in terms of both taxonomic affiliation and type of use, have been used traditionally in China. Medicine, forage and food are the three most important categories of use, the plants providing basic resources used by local people in their everyday lives. Local availability is the main factor influencing which species are used. Quantitative indexes, especially Cultural Value Index, proved very useful for evaluating the usefulness of plants as recorded in the literature.

## Background

Traditional knowledge of plants has played an important role in people’s lives historically and has the potential to continue to contribute much in the future for the sustainable development of societies and economies [1–3]. However, as with biodiversity, traditional knowledge is becoming endangered with the danger of being total loss [4, 5]. There are many causes of such endangerment, including changes occurring in the environment [6], urbanization and economic globalization [7, 8]. Urbanization is one of the most important factors globally causing loss of traditional knowledge [7, 8].

In contrast with terrestrial ecosystems, wetlands have been poorly studied ethnobotanically, even though, for people living in and around wetlands, wild wetland plants play important roles in their daily lives [9–12]. Plants are collected from wetlands for a wide variety of purposes, such as provision of medicine, food and building materials and to sell for cash income [9, 11, 13]. Wetlands are very susceptible to loss or degradation through urbanization [14–16], which can change their extent and species composition and lead to the loss of biodiversity [17–19]. Traditional knowledge about wetlands is declining along with wetland degradation and alteration [11, 12], an inevitable trend given the accelerating rate of urbanization that is now occurring.

Wetlands are widely distributed throughout China, but especially common in the east and south [20], where there are particularly rich traditions of local knowledge about the uses of their plants. There has been little ethnobotanical research specifically on wetland plants, but much information on traditional uses of wetland plants nevertheless does exist, though scattered through regional floras and other types of publication. Like traditional ethnobotanical knowledge generally, that concerned with wetland plants is becoming [11]. Ethnobotanical research on people’s knowledge of wetland plants in China is urgently needed.

Currently, most ethnobotanical research concerned with any habitat type (not just wetlands) is conducted on the basis of case studies undertaken at specific field locations. The results of such studies are important for understanding relationships between local people and their environments, including sometimes for providing guidance on the sustainable use of plants and their conservation. However, case studies unavoidably emphasize unique local features of the relationships between people and their environments [21]. There is a role for systematic reviews and meta-analyses on wider regional to international scales to investigate general patterns of knowledge and use relating to plants, including to provide contexts for local-level studies [21–25].

Quantitative methods have been successfully applied in ethnobotanical studies, especially in the evaluation of cultural value or importance of species [11, 24, 26]. However, most quantitative methods have been developed for the analysis of case studies based on field work. Which of these methods is most suitable for systematic reviews or meta-analyses remains little studied, with little published information available.

In this paper, we aim to answer the following questions through a study of the literature: 1. What are the botanical characteristics of wetland plants traditionally considered useful in China? 2. What are the main factors influencing the patterns of use of wetland plants in China? 3. Which of the available quantitative indexes is most suitable for evaluating traditional knowledge, as determined from the literature?

## Methods

### Data collection

Two criteria were used to identify the species included in this analysis. First, the species had to be wetland plants; we took the definition of a wetland as that given in the *Convention on Wetlands of International Importance especially as Waterfowl Habitat* (1971). Second, the species had to have been recorded as having traditional use. In this paper, we only paid attention on vascular plants. There have been very few systematic ethnobotanical studies conducted on wetlands in China. Most of the available ethnobotanical information on wetland plants is scattered sporadically through various publications, such as national and provincial floras, economic floras, and papers published in scientific journals. Our approach has been to identify, so far as we were able, all sources of potential information on wetland plants and then to search through this literature to compile an ethnobotanical inventory of wetland plants. Then we used the scientific names of the plants as key words to search further information on traditional use in the *China Science and Technology Journal Database*. The total number of principal literature sources studied was 56 [27–82].

Based on records in the literature, we classified uses into 11 groups: medicine, fodder, food, green manure, fiber, ornamental, liquor-making, environmental, industrial raw material, pesticide, and other. The medicine category includes plants used for treating animal as well as human diseases. Fodder refers to plants eaten by domestic animals. Edible plants are those as human food either in a raw or processed state. Green manure refers to plants employed as fertilizer. Fiber plants are those yielding fibers used by people; there are various ways in which they are extracted from the plants. Ornamental plants are those planted deliberately to beautify the environment. Liquor-making plants are those yielding either basic ingredients or supplementary materials used in making traditional liquor. The environmental category refers to plants used in soil conservation or the stabilization of dams. The industrial raw material category includes those plants providing raw materials for industrial production, such as for the manufacture of essential oils. Although not a typical traditional use, such plants can provide local people with sources of cash income and thus is important category of use for some people. The pesticide category refers to plants used for killing or driving away pests such as insects. Uses other than those in the above ten categories are grouped together in ‘other’.

### Data analysis

Use Value (UV) is a widely used statistic employed by ethnobotanists to provide a measure of the relative usefulness of plants to people [24, 83]. In this paper, we use the formula UV_i_ = ∑U_i_/n to calculate the use value of each species (i), U_i_ referring to the number of categories of use mentioned for a species in a particular literature source and n the total number of literature sources mentioning the species [23, 24, 84]. For example, if two literature sources (n = 2) mention species i, with three use categories mentioned in the first source and one in the second, then UV_i_ = (3 + 1)/2 = 2.

Family Use Value (FUV), a statistic developed by Phillips and Gentry [83], provides a measure of the relative usefulness of plant families. FUV for a particular family (j) is calculated using the formula FUV_j_ = ∑(UV_i_)/n, where UV_i_ is the use value of species i and n is the number of species in the family.

The statistic Relative Frequency of Citation (RFC_i_) is used as a measure of consensus between the information provided by different literature sources. RFC is similar conceptually to that of Utilization Frequency proposed by Ladio and Lozada [85]. RFC for a species is calculated as RFC_i_ = FC_i_/N [24], where FC_i_ is the number of literature sources mentioning species i and N the total number of literature sources consulted (N = 56 in the present case).

The cultural value (or importance value) of species in a given culture and the comparative importance of species interculturally are receiving growing attention in ethnobotanical studies, especially those concerned with medicinal plants [24, 25, 86, 87]. Here, measures of cultural value for wetland plant in China are provided by the statistics Cultural Importance Index (CI) and Cultural Value Index (CV), based on formulae given in Tardio & Pardo-de-Santayana and Reyes-Garcia et al. [24, 26].

Finally, regression analysis has been used to determine the relationships between RFC, CI and CV.

## Results

### Diversity of the useful wetland plants in China

A total of 350 wetland plant species (including 5 varieties), belonging to 66 families and 187 genera, were recorded as used in China according to the survey. The average number of species recorded per family was 5.3, with 15 families (22.7% of the total) having more species than the average (Table 1). The ten families (Poaceae, Polygonaceae, Cyperaceae, Lamiaceae, Asteraceae, Ranunculaceae, Hydrocharitaceae, Potamogetonaceae, Fabaceae, and Brassicaceae) contributed 58.6% of all species, the 5 with the highest number of species being Poaceae (46 species; 13.1% of the total), Polygonaceae (9.1%), Cyperaceae (8.3%), Lamiaceae (5.7%), and Asteraceae (5.1%), Twenty-five families (37.9% of the total) were represented by only one useful species each. The remaining 33 families contributed between 2 and 11 species each (0.6-3.1% of the total).

**Table 1:** **The taxonomic composition of wetland plants used traditionally and family use values** (**FUV**) **based on literature research**

Family	No. of genus (%)	No. of Species (%)	FUV
Poaceae	27 (14.4)	46 (13.1)	1.59
Polygonaceae	3 (1.6)	32 (9.1)	1.57
Cyperaceae	6 (3.2)	29 (8.3)	1.34
Lamiaceae	14 (7.5)	20 (5.7)	1.25
Asteraceae	14 (7.5)	18 (5.1)	1.30
Ranunculaceae	6 (3.2)	14 (4.0)	1.10
Hydrocharitaceae	6 (3.2)	13 (3.7)	1.51
Potamogetonaceae	1 (0.5)	12 (3.4)	1.33
Fabaceae	11 (5.9)	11 (3.1)	1.97
Brassicaceae	4 (2.1)	10 (2.9)	1.81
Apiaceae	5 (2.7)	7 (2.0)	1.38
Araceae	5 (2.7)	7 (2.0)	1.19
Rosaceae	3 (1.6)	7 (2.0)	1.56
Scrophulariaceae	6 (3.2)	7 (2.0)	1.05
Alismataceae	3 (1.6)	6 (1.7)	1.29
Chenopodiaceae	3 (1.6)	5 (1.4)	1.86
Commelinaceae	2 (1.1)	5 (1.4)	1.24
Eriocaulaceae	1 (0.5)	5 (1.4)	1.00
Primulaceae	2 (1.1)	5 (1.4)	1.24
Typhaceae	2 (1.1)	5 (1.4)	1.84
Urticaceae	4 (2.1)	5 (1.4)	1.51
Equisetaceae	1 (0.5)	4 (1.1)	1.21
Lemnaceae	3 (1.6)	4 (1.1)	1.44
Lythraceae	3 (1.6)	4 (1.1)	1.13
Onagraceae	2 (1.1)	4 (1.1)	1.04
Pontederiaceae	2 (1.1)	4 (1.1)	1.83
Trapaceae	1 (0.5)	4 (1.1)	1.75
Acanthaceae	3 (1.6)	3 (0.9)	1.00
Caryophllaceae	3 (1.6)	3 (0.9)	1.33
Nymphaeaceae	3 (1.6)	3 (0.9)	2.71
Plantaginaceae	1 (0.5)	3 (0.9)	1.23
Acoraceae	1 (0.5)	2 (0.6)	1.28
Amaranthaceae	1 (0.5)	2 (0.6)	2.82
Cannaceae	1 (0.5)	2 (0.6)	1.42
Haloragaceae	1 (0.5)	2 (0.6)	1.13
Lentibulariaceae	1 (0.5)	2 (0.6)	1.00
Menyanthaceae	1 (0.5)	2 (0.6)	2.06
Solanaceae	2 (1.1)	2 (0.6)	1.50
Valerianaceae	1 (0.5)	2 (0.6)	1.00
Verbenaceae	2 (1.1)	2 (0.6)	1.00
Violaceae	1 (0.5)	2 (0.6)	1.00
Amaryllidaceae	1 (0.5)	1 (0.3)	2.50
Apocynaceae	1 (0.5)	1 (0.3)	2.67
Azollaceae	1 (0.5)	1 (0.3)	2.22
Butomaceae	1 (0.5)	1 (0.3)	1.50
Cabombaceae	1 (0.5)	1 (0.3)	1.00
Campanulaceae	1 (0.5)	1 (0.3)	1.00
Ceratophyllaceae	1 (0.5)	1 (0.3)	1.71
Cucurbitaceae	1 (0.5)	1 (0.3)	3.20
Euphorbiaceae	1 (0.5)	1 (0.3)	1.00
Gentianaceae	1 (0.5)	1 (0.3)	1.00
Geraniaceae	1 (0.5)	1 (0.3)	1.00
Iridaceae	1 (0.5)	1 (0.3)	1.25
Juncaceae	1 (0.5)	1 (0.3)	1.71
Marsileaceae	1 (0.5)	1 (0.3)	1.71
Menispermaceae	1 (0.5)	1 (0.3)	1.00
Nelumbonaceae	1 (0.5)	1 (0.3)	2.22
Papaveraceae	1 (0.5)	1 (0.3)	1.00
Parkeriaceae	1 (0.5)	1 (0.3)	1.33
Penthoraceae	1 (0.5)	1 (0.3)	2.33
Phytolaccaceae	1 (0.5)	1 (0.3)	2.75
Plumbaginaceae	1 (0.5)	1 (0.3)	1.00
Salviniaceae	1 (0.5)	1 (0.3)	1.89
Saururaceae	1 (0.5)	1 (0.3)	1.33
Saxifragaceae	1 (0.5)	1 (0.3)	1.67
Schizaeaceae	1 (0.5)	1 (0.3)	1.50

The family names on the list are arranged in the order of the descending number of species.

Some taxa were obviously dominant at the generic level, 32 genera (17.1% of the total) being represented by 3 or more species. The top scorer was *Polygonum* (24 species), followed by: *Potamogeton* (12); *Cyperus* (10); *Scirpus* and *Rumex* (both 7); *Ranunculus* (6); *Carex*, *Eriocaulon*, *Echinochloa*, *Cardamine*, and *Potentilla* (all 5); *Blyxa*, *Bromus*, *Eleocharis*, *Equisetum*, *Lysimachia*, *Najas*, *Paspalum*, *Stachys*, *Trapa*, and *Typha* (all 4); and then *Alisma*, *Arisaema*, *Clematis*, *Leersia*, *Ludwigia*, *Miscanthus*, *Monochoria*, *Murdannia*, *Oenanthe*, *Plantago*, and *Rorippa* (all 3). The dominant genera belonged to the same families as scored highest at the family level, for example Polygonaceae, Cyperaceae, Potamogetonaceae, Poaceae, Hydrocharitaceae and Ranunculaceae.

Scores for Family Use Value (FUV) fell between 1 (for 14 families) and 3.2 (Cucurbitaceae) (Table 1). The top 10 families according to this measure (all with FUV >2.0) were completely different from those scoring highly according to number of species. There was no obvious correlation between FUV and number of species used per family. All top 10 families based on FUV were families with few wetland species (3 or fewer). However, there were also families having few species with low FUV scores.

### Characteristics of traditional use of wetland plants

Medicine, fodder and food were the main uses made of wetland plants according to number of species (Table 2). Seventy percent of all species were recorded to be of medicinal use, nearly half of were employed as forage and somewhat fewer as food. Fewer plants were recorded as employed for green manure, fiber, or as sources of raw materials for industry, but all these were noticeably important types of use. The other five categories of use accounted for only a small proportion of total uses. Twenty-six species (7.4% of all species) provided insecticides and 22 species (6.3%) were employed in the making of liquor. Several plants were sold for cash, such as species of *Polygonum*, among others. The ‘Other’ category included some plants used for skin care, such as *Coix lacryma*-*jobi* and *Zizania latifolia*, and others in house construction, such as *Arundo donax*, *Miscanthus sacchariflorus*, and *Phragmites australis*. Although few species were included in the construction category, nevertheless wetland plants used in construction can be of major importance to local people.

**Figure 1:**
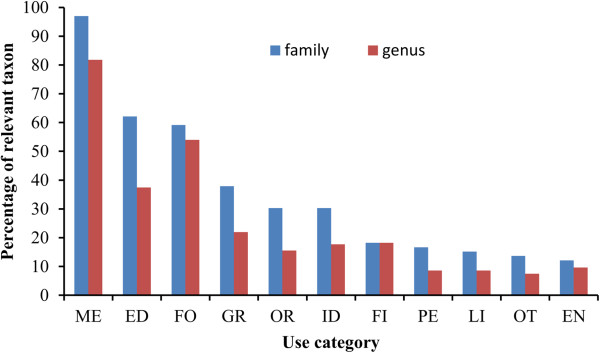
**The percentage of relevant families and genera for each use category.** EN = environmental use; PE = pesticide; OT = other; LI = liquor–making material; ID = industrial raw material; FI = fiber; OR = ornamental; GR = green manure; ED = food; FO = fodder; ME = medicine.

Different families made very different contribution to different use categories (Figure 1). Over half of the families contributed to the top three categories that were medicine (97% of families), food (62.1%) and forage (59.1%); about one-third contributed to each of green manure, ornamental, and industrial use. However, other categories of use were more obviously concentrated within certain families. For example, fewer than 20% of families contributed to fiber use, pesticides, liquor–making, or environmental use. Nearly half of species providing pesticides were in the Polygonaceae and 54.2% of those used for environmental protection (such as preventing soil erosion and stabilizing dams) in the Poaceae. Species of the Poaceae and Cyperaceae contributed greatly to the fiber group (63.5% of all species so used), while those in the Polygonaceae, Poaceae, and Trapaceae were well represented in liquor-making (54% of species used). Genera showed similar patterns to those shown by families. The results as a whole showed that the top three use categories of medicine, food and forage made use of a broader spectrum of plants taxonomically than other uses.

**Table 2:** **Use categories of wetland plants and the numbers of related species**

Type of use	No. of species	Percentage of the total (%)
Medicine	263	75.1
Forage	173	49.4
Food	101	28.9
Green manure	53	15.1
Fiber	52	14.9
Industrial raw material	48	13.7
Ornamental	32	9.1
Pesticide	26	7.4
Environmental use	24	6.9
Liquor –making material	22	6.3
Other	15	4.3

Some of the top families contributed greatly to some of the use categories (Table 3). The top ten families contributed about half of all species used medicinally, over 66% of those providing fodder (though lacking any contribution from Ranunculaceae) and nearly fifty percent of those used as food. Seven of the top ten families contributed 52.8% of species used as green manure. The top ten families together contributed 73% of species used for fiber, although actually only three (Poaceae, Cyperaceae, and Fabaceae) made substantial contributions. Similar patterns were apparent in the other use categories. Some top families, such as Lamiaceae, Hydrocharitaceae, Potamogetonaceae, and Brassicaceae, contributed only to certain of the major categories of use, for example Rancunculaceae (one of the top ten families) was only used for medicine, food and pesticide. Thus, families with large numbers of species used did not necessarily contribute to all categories of use.

**Table 3:** **Contributions of the top 10 families** (**in terms of numbers of species**) **to different use categories**

Family	ME (%)	FO (%)	ED (%)	GR (%)	FI (%)	ID (%)	OR (%)	PE (%)	EN (%)	LI (%)	OT (%)
Poaceae	14 (5.3)	42 (24.3)	7 (6.9)	2 (3.8)	18 (34.6)	2 (4.2)	4 (12.5)	0 (0.0)	13 (54.2)	4 (18.2)	6 (40.0)
Polygonaceae	32 (12.2)	11 (6.4)	11 (10.9)	1 (1.9)	0 (0.0)	14 (29.2)	1 (3.1)	12 (46.2)	1 (4.2)	5 (22.7)	1 (6.7)
Cyperaceae	17 (6.5)	19 (11.0)	3 (3.0)	1 (1.9)	15 (28.8)	1 (2.1)	2 (6.3)	0 (0.0)	2 (8.3)	2 (9.1)	0 (0.0)
Lamiaceae	20 (7.6)	2 (1.2)	5 (5.0)	0 (0.0)	0 (0.0)	4 (8.3)	0 (0.0)	1 (3.8)	0 (0.0)	0 (0.0)	0 (0.0)
Asteraceae	13 (4.9)	6 (3.5)	7 (6.9)	2 (3.8)	0 (0.0)	1 (2.1)	0 (0.0)	1 (3.8)	1 (4.2)	1 (4.5)	0 (0.0)
Ranunculaceae	13 (4.9)	0 (0.0)	1 (1.0)	0 (0.0)	0 (0.0)	0 (0.0)	0 (0.0)	5 (19.2)	0 (0.0)	0 (0.0)	0 (0.0)
Hydrocharitaceae	3 (1.1)	12 (6.9)	3 (3.0)	7 (13.2)	0 (0.0)	0 (0.0)	1 (3.1)	0 (0.0)	0 (0.0)	0 (0.0)	0 (0.0)
Potamogetonaceae	5 (1.9)	10 (5.8)	1 (1.0)	7 (13.2)	0 (0.0)	0 (0.0)	0 (0.0)	0 (0.0)	0 (0.0)	0 (0.0)	0 (0.0)
Fabaceae	9 (3.4)	8 (4.6)	1 (1.0)	8 (15.1)	5 (9.6)	2 (4.2)	0 (0.0)	0 (0.0)	4 (16.7)	0 (0.0)	0 (0.0)
Brassicaceae	8 (3.0)	5 (2.9)	8 (7.9)	0 (0.0)	0 (0.0)	5 (10.4)	1 (3.1)	0 (0.0)	0 (0.0)	0 (0.0)	0 (0.0)
Total	134 (51.0)	115 (66.5)	47 (46.5)	28 (52.8)	38 (73.0)	29 (60.4)	9 (28.1)	19 (73.1)	21 (87.5)	12 (54.5)	7 (46.7)

Note: *ME* = medicine; *ED* = food; *FO* = fodder; *GR* = green manure; *OR* = ornamental; *ID* = industrial raw material; *FI* = fiber; *PE* = pesticide; *LI* = liquor–making material; *OT* = other; *EN* = environmental use.

### Use value of wetland plants

The Use Values (UV) of species are shown on Table 4. UV varies between 1.0 and 3.71, with ten species having UV ≥ 3.00. *Phragmites australis*, which was highest scoring (UV = 3.71), is one of the dominant species of wetland plant communities in China and distributed widely in many parts of the country. At least seven types of use for this species are mentioned frequently in the literature. *Glycine soja* was second in rank order (UV = 3.5), followed by *Zizania latifolia* and *Rorippa islandica* (both UV = 3.33), *Actinostemma tenerum* (UV = 3.2), *Rumex acetosa* (UV = 3.17) and *Nymphaea tetragona* (UV = 3.13). Among species with a UV value of 3.00, *Euryale ferox* was recorded in ten literature sources, *Saccharum spontaneum* in three and *Oenanthe sinensis* in one; all are plants with multiple uses. There were 165 species (about 47% of the total) with the lowest possible score (UV = 1.0). Among these, one hundred and thirty-nine species (84.2%) had only one type of use and twenty-two species (13.3%) had two.

**Table 4:** **Ethnobotanical inventory and some quantitative indexes of useful wetland plants in China**

**Species**	**UV**	**RFC**	**CI**	**CV**	**Use**	**Reference(s)**
*Acorus calamus* L.	1.55	0.20	0.30	0.03795	ED,ME,FI,FO,PE,OR,ID	[27–29, 45, 46, 59, 63, 65, 68, 62, 67]
*Acorus gramineus* Aiton	1.00	0.11	0.11	0.00209	ED,ME	[27, 44, 46, 59, 63, 78]
*Actinostemma tenerum* Griff.	3.20	0.09	0.29	0.01160	ED,ME,FO,GR,ID	[27–29, 58, 64]
*Adenostemma lavenia* (L.) Kuntze	1.00	0.07	0.07	0.00046	ED	[27, 29, 46, 58]
*Aeginetia indica* L.	1.00	0.05	0.05	0.00026	ME	[28, 29, 46]
*Aeschynomene indica* L.	2.00	0.05	0.11	0.00157	ME,GR,FI	[29, 46, 58]
*Ageratum conyzoides* L.	2.00	0.05	0.11	0.00209	ME,FO,GR,EN	[28, 29, 46]
*Ajuga ciliata* Bunge	1.00	0.05	0.05	0.00026	ME	[28, 29, 46]
*Ajuga multiflora* Bunge	1.00	0.04	0.04	0.00012	ME	[28, 29]
*Alisma canaliculatum* A. Braun & C. D. Bouché	1.50	0.04	0.05	0.00035	ME,OR	[29, 46]
*Alisma gramineum* Lej.	1.00	0.02	0.02	0.00003	ME	[64]
*Alisma plantago-aquatica* L.	1.13	0.14	0.16	0.00417	ME,OR	[27–29, 45, 46, 59, 64, 65]
*Alternanthera philoxeroides* (Mart.) Griseb.	2.83	0.11	0.30	0.01183	ME,GR,FO,EN	[27–29, 46, 57, 58]
*Alternanthera sessilis* (L.) DC.	2.80	0.09	0.25	0.00812	ED,ME,FO,GR	[27, 29, 46, 58, 62]
*Amethystea coerulea* L.	1.00	0.04	0.04	0.00012	ME	[28, 29]
*Ammannia baccifera* L.	1.00	0.04	0.04	0.00023	ME,FO	[46, 62]
*Amphicarpaea trisperma* Baker	1.00	0.02	0.02	0.00003	FO	[28]
*Anemone hupehensis* (Lemoine) Lemoine	1.67	0.05	0.09	0.00087	ME,PE	[28, 29, 46]
*Apium leptophyllum* (Pers.) F. Muell.	1.00	0.02	0.02	0.00003	FO	[28]
*Apocynum venetum* L.	2.67	0.05	0.14	0.00278	ED,ME,FI,ID	[28, 29, 46]
*Arisaema amurense* Maxim.	1.00	0.07	0.07	0.00046	ME	[27, 28, 46, 59]
*Arisaema du-bois-reymondiae* Engl.	1.00	0.02	0.02	0.00003	ME	[29]
*Arisaema heterophyllum* Blume	1.00	0.02	0.02	0.00003	ME	[69]
*Artemisia capillaris* Thunb.	2.20	0.09	0.20	0.00957	ED,ME,FO,LI,PE,ID	[27–29, 58, 67]
*Artemisia selengensis* Turcz. ex Besser	1.60	0.09	0.14	0.00348	ED,ME,FO	[27, 29, 46, 65, 70]
*Arthraxon hispidus* (Thunb.) Makino	1.50	0.07	0.11	0.00209	ME,FI,FO	[28, 46, 57, 67]
*Arundinella anomala* Steud.	2.00	0.04	0.07	0.00046	FI,FO	[28, 29]
*Arundo donax* L.	2.63	0.14	0.38	0.02922	ME,FI,FO,OR,EN,OT	[27–29, 36, 37, 45, 46, 59]
*Arundo donax* var. *versicolor* (Mill.) Stokes	1.00	0.04	0.04	0.00012	OR	[27, 38]
*Astilbe chinensis* Franch. & Sav.	1.67	0.05	0.09	0.00130	ME,OR,ID	[28, 29, 46]
*Astragalus adsurgens* Pall.	1.67	0.05	0.09	0.00130	ME,FO,EN	[28, 29, 46]
*Atropanthe sinensis* Pascher	1.67	0.05	0.09	0.00087	ME,ID	[28, 29, 46]
*Azolla imbricata* (Roxb.) Nakai	2.22	0.16	0.36	0.02087	ME,FO,GR,PE	[27–29, 31, 32, 46, 58, 59, 62]
*Bacopa monnieri* (L.) Wettst.	1.00	0.04	0.04	0.00012	ME	[28, 29]
*Beckmannia syzigachne* (Steud.) Fernald	1.67	0.05	0.09	0.00130	ED,ME,FO	[28, 57, 68]
*Berteroa incana* DC.	1.00	0.02	0.02	0.00003	ID	[28]
*Bidens parviflora* Willd.	1.33	0.05	0.07	0.00070	ED,ME	[28, 29, 46]
*Bidens tripartita* L.	1.00	0.05	0.05	0.00026	ME	[28, 29, 46]
*Blyxa aubertii* Rich.	1.00	0.02	0.02	0.00003	FO	[28]
*Blyxa echinosperma* (C. B. Clarke) Hook. f.	1.00	0.04	0.04	0.00012	FO	[27, 28]
*Blyxa japonica* Maxim. ex Asch. & Gürke	1.00	0.02	0.02	0.00003	FO	[28]
*Blyxa leiosperma* Koidz.	1.00	0.02	0.02	0.00003	FO	[27]
*Boehmeria gracilis* C. H. Wright	1.67	0.05	0.09	0.00130	ME,FI,ID	[28, 29, 46]
*Brasenia schreberi* J. F. Gmel.	1.00	0.05	0.05	0.00052	ED,ME	[27, 29, 46]
*Bromus catharticus* Vahl	1.00	0.04	0.04	0.00012	FO	[27, 29]
*Bromus inermis* Leyss.	2.50	0.04	0.09	0.00087	ED,FO,EN	[27, 28]
*Bromus japonicus* Thunb.	1.83	0.11	0.20	0.00957	ED,ME,FO,FI,LI	[27, 28, 45, 46, 57, 62]
*Bromus remotiflorus* (Steud.) Ohwi	1.33	0.05	0.07	0.00070	FI,FO	[45, 57, 62]
*Butomus umbellatus* L.	1.50	0.04	0.05	0.00035	FI,OR	[28, 64]
*Calamagrostis epigeios* (L.) Roth	2.60	0.09	0.23	0.00754	FI,FO,OT,EN	[27–29, 57, 67]
*Calamagrostis pseudophragmites* (Hall. f.) Koel.	2.00	0.07	0.14	0.00186	FO,EN	[27–29, 57]
*Caldesia reniformis* Makino	1.00	0.02	0.02	0.00003	OR	[29]
*Caltha palustris* L.	1.33	0.05	0.07	0.00104	ED,ME,PE	[29, 68, 82]
*Canna generalis* L. H. Bailey	1.50	0.04	0.05	0.00035	FI,OR	[27, 29]
*Canna indica* L.	1.33	0.05	0.07	0.00139	ED,ME,FI,FO	[27, 46, 62]
*Capillipedium parviflorum* (R. Br.) Stapf	1.00	0.02	0.02	0.00003	FO	[28]
*Cardamine flexuosa* With.	1.00	0.05	0.05	0.00026	ME	[28, 29, 58]
*Cardamine impatiens* L.	1.75	0.07	0.13	0.00244	ED,ME,FO	[27–29, 63]
*Cardamine leucantha* (Tausch) O. E. Schulz	1.25	0.07	0.09	0.00116	ED,ME	[28, 29, 46, 70]
*Cardamine lyrata* Bunge	1.75	0.07	0.13	0.00162	ED,ME	[27–29, 46]
*Cardamine macrophylla* Willd.	1.67	0.05	0.09	0.00130	ED,ME,FO	[29, 81, 82]
*Carex baccans* Nees	1.00	0.04	0.04	0.00023	ED,ME	[28, 46]
*Carex dispalata* Boott	1.50	0.04	0.05	0.00035	FI,FO	[28, 67]
*Carex leiorhyncha* C. A. Mey.	1.00	0.04	0.04	0.00012	FO	[28, 67]
*Carex scabrifolia* Steud.	1.00	0.04	0.04	0.00012	FI	[28, 29]
*Carex tangiana* Ohwi	1.50	0.04	0.05	0.00035	FO,OR	[28, 67]
*Catabrosa aquatica* P. Beauv.	1.00	0.02	0.02	0.00003	FO	[28]
*Centaurium meyeri* Druce	1.00	0.04	0.04	0.00012	ME	[28, 46]
*Centipeda minima* (L.) A. Braun & Asch.	1.00	0.07	0.07	0.00046	ME	[28, 29, 46, 78]
*Ceratophyllum demersum* L.	1.71	0.13	0.21	0.00487	ME,FO	[27–29, 46, 58, 63, 64]
*Ceratopteris thalictroides* (L.) Brongn.	1.33	0.11	0.14	0.00278	ED,ME	[27–29, 46, 58, 63]
*Chenopodium ambrosioides* L.	2.20	0.09	0.20	0.00638	ME,ED,PE,ID	[27–29, 46, 58]
*Chenopodium serotinum* L.	1.00	0.04	0.04	0.00023	ME,FO	[46, 62]
*Cicuta virosa* L.	1.00	0.04	0.04	0.00012	ME	[28, 46]
*Clematis cadmia* Buch.-Ham. ex Hook.f. & Thomson	1.00	0.04	0.04	0.00012	ME	[28, 46]
*Clematis finetiana* H. Lév. & Vaniot	1.00	0.05	0.05	0.00026	ME	[28, 29, 46]
*Clematis orientalis* L.	1.00	0.02	0.02	0.00003	ME	[28]
*Clinopodium chinense* Kuntze	1.00	0.04	0.04	0.00012	ME	[28, 46]
*Clinopodium gracile* (Bentham) Matsumura	1.00	0.05	0.05	0.00026	ME	[28, 29, 46]
*Cnidium monnieri* (L.) Cuss.	1.33	0.05	0.07	0.00070	ME,FO	[28, 29, 46]
*Coix lacryma-jobi* L.	2.30	0.18	0.41	0.04000	ED,ME,FI,FO,LI,OT	[27, 41, 42, 45, 46, 59, 62, 63, 65, 71]
*Colocasia esculenta* (L.) Schott	1.83	0.11	0.20	0.00574	ED,ME,FO	[27, 29, 46, 65, 59, 62]
*Commelina benghalensis* L.	1.00	0.07	0.07	0.00093	ME,OR	[27–29, 46]
*Commelina communis* L.	1.20	0.09	0.11	0.00261	ED,ME,FO	[27–29, 46, 70]
*Corydalis racemosa* Pers.	1.00	0.07	0.07	0.00046	ME	[28, 29, 46, 58]
*Crotalaria assamica* Benth.	2.00	0.04	0.07	0.00093	ME,FI,FO,GR	[28, 29]
*Crypsis aculeata* Aiton	1.50	0.04	0.05	0.00035	FO,EN	[28, 29]
*Cyperus compressus* L.	1.00	0.04	0.04	0.00012	FO	[62, 67]
*Cyperus difformis* L.	1.00	0.11	0.11	0.00209	ME,FI	[28, 46, 62–64, 67]
*Cyperus exaltatus* Retz.	1.00	0.07	0.07	0.00093	FI,FO	[28, 29, 62, 67]
*Cyperus glomeratus* L.	1.60	0.09	0.14	0.00464	ME,FO,FI,GR	[28, 46, 62, 64, 67]
*Cyperus imbricatus* Retz.	1.00	0.04	0.04	0.00012	FI	[28, 29]
*Cyperus iria* L.	1.00	0.05	0.05	0.00052	ME,FO	[46, 62, 67]
*Cyperus michelianus* (L.) Link	1.00	0.02	0.02	0.00003	ME	[46]
*Cyperus microiria* Steud.	1.00	0.02	0.02	0.00003	FO	[62]
*Cyperus pilosus* Vahl	1.00	0.07	0.07	0.00139	ME,FI,FO	[27, 45, 62, 63]
*Cyperus pygmaeus* Rottb.	1.00	0.02	0.02	0.00003	FO	[62]
*Dichrocephala auriculata* Druce	1.00	0.05	0.05	0.00026	ME	[28, 29, 46]
*Dichrocephala benthamii* C. B. Clarke	1.00	0.05	0.05	0.00026	ME	[28, 29, 46]
*Dicliptera chinensis* (L.) Juss.	1.00	0.05	0.05	0.00026	ME	[28, 29, 46]
*Duchesnea indica* (Andrews) Focke	1.20	0.09	0.11	0.00261	ED,ME,PE	[28, 29, 46, 69, 78]
*Echinochloa caudata* Roshev.	1.00	0.02	0.02	0.00003	FO	[28]
*Echinochloa crus-galli* (L.) P. Beauv.	2.40	0.09	0.21	0.01044	ED,ME,FI,FO,GR,LI	[28, 45, 46, 57, 62]
*Echinochloa crus-galli* var. *mitis* (Pursh) Peterm.	1.00	0.02	0.02	0.00003	FO	[62]
*Echinochloa crus-galli* var. *zelayensis* (Kunth) Hitchc.	1.00	0.02	0.02	0.00003	FO	[62]
*Echinochloa crus-pavonis* (Kunth) Schult.	1.00	0.02	0.02	0.00003	FO	[62]
*Eichhornia crassipes* (Mart.) Solms	2.67	0.11	0.29	0.01670	ED,ME,FO,OR,GR,EN	[27–29, 46, 57, 62]
*Eleocharis dulcis* Trin. ex Henschel.	2.00	0.11	0.21	0.00626	ED,ME,FO	[27, 29, 43, 62, 65, 73]
*Eleocharis plantagineiformis* Tang & F. T. Wang	1.00	0.02	0.02	0.00003	FO	[28]
*Eleocharis valleculosa* Ohwi	1.33	0.05	0.07	0.00070	FI,FO	[27, 57, 62]
*Eleocharis yokoscensis* (Franch. & Savat.) Tang & F. T. Wang	1.00	0.02	0.02	0.00003	ME	[46]
*Elsholtzia kachinensis* Prain	1.40	0.09	0.13	0.00304	ED,ME,FO	[28, 29, 46, 63, 72]
*Equisetum debile* Roxb. ex Vaucher	1.50	0.07	0.11	0.00139	ME,OT	[27, 28, 46, 58]
*Equisetum hyemale* L.	1.33	0.05	0.07	0.00070	ME,OT	[28, 46, 67]
*Equisetum pratense* Ehrh.	1.00	0.04	0.04	0.00012	ME	[28, 67]
*Equisetum ramosissimum* Desf.	1.00	0.05	0.05	0.00026	ME	[27, 28, 65]
*Eriocaulon australe* R. Br.	1.00	0.04	0.04	0.00012	ME	[28, 46]
*Eriocaulon buergerianum* Körn.	1.00	0.11	0.11	0.00104	ME	[27–29, 45, 46, 59]
*Eriocaulon cinereum* R. Br.	1.00	0.04	0.04	0.00012	ME	[28, 63]
*Eriocaulon decemflorum* Maxim.	1.00	0.02	0.02	0.00003	ME	[27]
*Eriocaulon robustius* Makino	1.00	0.02	0.02	0.00003	ME	[28]
*Euphorbia thymifolia* L.	1.00	0.07	0.07	0.00046	ME	[28, 29, 46, 68]
*Euryale ferox* Salisb.	3.00	0.18	0.54	0.06088	ED,ME,FO,GR,LI,OR,ID	[27, 29, 45, 46, 57, 58, 62–65]
*Fimbristylis miliacea* (L.) Vahl	1.67	0.05	0.09	0.00130	ME,FI,FO	[27, 28, 46]
*Geranium sibiricum* L.	1.00	0.04	0.04	0.00012	ME	[28, 46]
*Geum aleppicum* Jacq.	1.75	0.07	0.13	0.00244	ED,ME,ID	[28, 29, 46, 67]
*Glaux maritima* L.	1.00	0.04	0.04	0.00012	ED	[28, 70]
*Glechoma longituba* (Nakai) Kuprian.	1.00	0.07	0.07	0.00046	ME	[28, 29, 46, 63]
*Glycine soja* Siebold & Zucc.	3.50	0.07	0.25	0.00974	ED,ME,FI,FO,GR,EN	[28, 29, 57, 67]
*Glycyrrhiza pallidiflora* Maxim.	1.00	0.05	0.05	0.00078	ME,FI,GR	[28, 29, 46]
*Halerpestes cymbalaria* Greene	1.00	0.02	0.02	0.00003	ME	[28]
*Halerpestes ruthenica* (Jacq.) Ovcz.	1.00	0.02	0.02	0.00003	PE	[28]
*Hemarthria altissima* (Poir.) Stapf & C. E. Hubb.	1.50	0.04	0.05	0.00035	FO,FI	[27, 28]
*Hemarthria compressa* (L. f.) R. Br.	1.00	0.02	0.02	0.00003	FO	[28]
*Hydrilla verticillata* (L. f.) Royle	2.00	0.05	0.11	0.00104	FO,GR	[27, 28, 64]
*Hydrocharis dubia* (Blume) Backer	1.67	0.05	0.09	0.00130	ED,FO,GR	[27–29]
*Hygrophila salicifolia* (Vahl) Nees	1.00	0.04	0.04	0.00012	ME	[28, 46]
*Inula japonica* Thunb.	1.00	0.05	0.05	0.00026	ME	[28, 29, 46]
*Iris tectorum* Maxim.	1.25	0.07	0.09	0.00116	ME,OR	[27, 29, 46, 59]
*Ixeris japonica* Nakai	1.00	0.04	0.04	0.00012	ME	[28, 46]
*Ixeris polycephala* Cass.	1.00	0.02	0.02	0.00003	ME	[29]
*Juncus effusus* L.	1.71	0.13	0.21	0.00731	ME,FI,OT	[27–29, 46, 64, 65, 67]
*Kyllinga brevifolia* Rottb.	1.00	0.04	0.04	0.00012	ME	[28, 46]
*Kyllinga colorata* (L.) Druce	1.00	0.02	0.02	0.00003	ME	[28]
*Lactuca tatarica* C. A. Mey.	2.00	0.02	0.04	0.00012	ED,FO	[28]
*Lagedium sibiricum* (L.) Soják	1.00	0.02	0.02	0.00003	ED	[70]
*Lamium amplexicaule* L.	1.00	0.05	0.05	0.00026	ME	[28, 29, 79]
*Lamium barbatum* Siebold & Zucc.	1.00	0.05	0.05	0.00026	ME	[28, 29, 46]
*Lapsana apogonoides* Maxim.	1.00	0.05	0.05	0.00026	FO	[28, 29, 57]
*Leersia hexandra* Sw.	1.50	0.04	0.05	0.00052	ME,FO,ID	[28, 46]
*Leersia japonica* Makino	1.00	0.02	0.02	0.00003	ME	[46]
*Leersia oryzoides* (L.) Sw.	1.00	0.02	0.02	0.00003	FO	[28]
*Lemna minor* L.	1.75	0.07	0.13	0.00244	ME,FO,GR	[28, 48, 59, 62]
*Lemna trisulca* L.	1.00	0.02	0.02	0.00003	FO	[28]
*Leptochloa chinensis* (L.) Nees	1.00	0.05	0.05	0.00026	FO	[27, 29, 62]
*Limonium sinense* Kuntze	1.00	0.05	0.05	0.00026	ME	[28, 29, 46]
*Lobelia chinensis* Lour.	1.00	0.07	0.07	0.00046	ME	[28, 29, 46, 58]
*Lotus tenuis* Waldst. & Kit. ex Willd.	1.00	0.04	0.04	0.00012	ME	[28, 46]
*Ludwigia adscendens* (L.) H. Hara	1.17	0.11	0.13	0.00244	ME,FO	[27–29, 46, 58, 62]
*Ludwigia hyssopifolia* (G. Don) Exell	1.00	0.05	0.05	0.00026	ME	[28, 29, 46]
*Ludwigia prostrata* Roxb.	1.00	0.05	0.05	0.00052	ME,FO	[27, 46, 62]
*Lycopus lucidus* Turcz.	1.20	0.09	0.11	0.00174	ED,ME	[28, 29, 46, 69, 70]
*Lycoris radiata* (L'Hér.) Herb.	2.50	0.07	0.18	0.00812	ED,ME,PE,LI,FI,OT,ID	[27–29, 46]
*Lygodium japonicum* (Thunb.) Sw.	1.50	0.07	0.11	0.00209	ME,PE,ED	[28, 29, 46, 77]
*Lysimachia christinae* Hance	1.00	0.09	0.09	0.00072	ME	[27–29, 46, 58]
*Lysimachia congestiflora* Hemsl.	1.00	0.07	0.07	0.00046	ME	[27–29, 46]
*Lysimachia fortunei* Maxim.	1.20	0.09	0.11	0.00174	ME,FO	[27–29, 46, 58]
*Lysimachia heterogenea* Klatt	2.00	0.02	0.04	0.00012	ME,GR	[28]
*Lythrum salicaria* L.	1.50	0.14	0.21	0.01113	ME,OR,FO,ID	[27–29, 46, 58, 62, 64, 65]
*Marsilea quadrifolia* L.	1.71	0.13	0.21	0.00974	ED,ME,FO,GR	[27–29, 46, 56, 58, 59]
*Mazus japonicus* (Thunb.) Kuntze	1.00	0.04	0.04	0.00012	ME	[27, 46]
*Melilotus indicus* (L.) All.	2.33	0.05	0.13	0.00244	ME,FO,GR,EN	[28, 46, 57]
*Mentha haplocalyx* Briq.	1.67	0.21	0.36	0.02087	ED,ME,ID	[27–29, 45, 46, 58, 59, 65, 67, 70, 78, 77]
*Microstegium ciliatum* A. Camus	1.50	0.04	0.05	0.00035	FI,FO	[28, 29]
*Mimulus tenellus* Bunge	1.00	0.05	0.05	0.00052	ED,ME	[28, 29, 46]
*Miscanthus floridulus* Warb. ex K. Schum. & Lauterb.	1.83	0.11	0.20	0.00765	ME,FO,FI,EN	[27–29, 46, 57, 62]
*Miscanthus sacchariflorus* (Maxim.) Hack.	2.00	0.09	0.18	0.00435	FI,FO,EN	[27, 28, 45, 57, 62]
*Miscanthus sinensis* Andersson	2.00	0.11	0.21	0.00835	ME,FI,FO,EN	[27–29, 45, 46, 62]
*Monochoria hastata* (L.) Solms	1.00	0.04	0.04	0.00012	ED	[28, 56]
*Monochoria korsakowii* Regel & Maack	2.00	0.14	0.29	0.01855	ED,ME,FO,OR,GR	[27–29, 46, 56, 57, 62, 64]
*Monochoria vaginalis* (Burm. f.) C. Presl ex Kunth	1.67	0.11	0.18	0.00696	ED,ME,FO,GR	[27, 46, 56, 57, 62, 70]
*Mosla dianthera* (Buch.-Ham. ex Roxb.) Maxim.	1.33	0.05	0.07	0.00070	ME,PE	[28, 29, 46]
*Murdannia keisak* (Hassk.) Hand.-Mazz.	1.00	0.02	0.02	0.00003	FO	[28]
*Murdannia nudiflora* (L.) Brenan	1.00	0.04	0.04	0.00012	ME	[28, 29]
*Murdannia triquetra* G. Brückn.	2.00	0.07	0.14	0.00278	ME,ED,FO	[27, 29, 46, 49]
*Myosoton aquaticum* Moench	2.00	0.05	0.11	0.00157	ME,ED,FO	[28, 29, 46]
*Myriophyllum spicatum* L.	1.25	0.07	0.09	0.00116	ME,FO	[27–29, 64]
*Myriophyllum verticillatum* L.	1.00	0.05	0.05	0.00026	FO	[27, 28, 64]
*Najas foveolata* A. Braun ex Magnus	2.00	0.02	0.04	0.00012	FO,GR	[28]
*Najas graminea* Delile	2.00	0.04	0.07	0.00046	FO,GR	[28, 29]
*Najas marina* L.	1.50	0.07	0.11	0.00139	FO,GR	[27, 28, 62, 64]
*Najas minor* All.	1.50	0.07	0.11	0.00139	FO,GR	[27, 28, 62, 64]
*Nanocnide japonica* Blume	1.00	0.05	0.05	0.00026	ME	[27, 28, 46]
*Nanocnide lobata* Wedd.	1.33	0.05	0.07	0.00070	ME,GR	[28, 29, 46]
*Nasturtium officinale* R. Br.	2.25	0.07	0.16	0.00417	ED,ME,OR,ID	[28, 46, 58, 65]
*Nelumbo nucifera* Gaertn.	2.22	0.16	0.36	0.02087	ED,ME,OR,FO	[27, 29, 45, 58, 46, 59, 62, 65, 66]
*Nepeta cataria* L.	1.50	0.07	0.11	0.00139	ME,ID	[28, 29, 46, 66]
*Nuphar pumila* (Timm) DC.	2.00	0.13	0.25	0.01136	ME,ED,OR,FO	[27, 29, 46, 54, 58, 59, 62]
*Nymphaea tetragona* Georgi	3.13	0.14	0.45	0.02899	ED,LI,ME,OR,GR	[27, 29, 45, 46, 58, 62, 64, 65]
*Nymphoides indica* (L.) Kuntze	2.00	0.02	0.04	0.00012	FO,GR	[27]
*Nymphoides peltata* (S. G. Gmel.) Kuntze	2.13	0.14	0.30	0.01971	ME,FO,GR,OR,ED	[27–29, 46, 58, 59, 62, 70]
*Oenanthe benghalensis* Benth. & Hook.f.	1.00	0.04	0.04	0.00012	ME	[28, 46]
*Oenanthe javanica* DC.	1.36	0.25	0.34	0.02313	ED,ME,FO	[27–29, 45, 46, 56–58, 64–66, 72, 70, 76]
*Oenanthe sinensis* Dunn	3.00	0.02	0.05	0.00026	ED,ME,FO	[28]
*Oenothera rosea* Aiton	1.00	0.04	0.04	0.00012	ME	[28, 29]
*Origanum vulgare* L.	2.00	0.05	0.11	0.00157	ME,ID,LI	[28, 29, 46]
*Ottelia acuminata* (Gagnep.) Dandy	1.00	0.04	0.04	0.00023	ED,ME	[65, 75]
*Ottelia alismoides* (L.) Pers.	2.60	0.09	0.23	0.00942	ED,ME,OR,GR,FO	[27–29, 46, 59]
*Panicum paludosum* Roxb.	1.00	0.02	0.02	0.00003	FO	[28]
*Paspalum dilatatum* Poir.	1.00	0.05	0.05	0.00026	FO	[29, 40, 62]
*Paspalum distichum* L.	1.00	0.04	0.04	0.00023	FO,EN	[27, 62]
*Paspalum paspaloides* Scribn.	2.00	0.02	0.04	0.00012	FO,EN	[28]
*Paspalum thunbergii* Kunth ex Steud.	1.00	0.04	0.04	0.00012	FO	[57, 62]
*Penthorum chinense* Pursh	2.33	0.05	0.13	0.00244	ED,ME,FO,GR	[28, 29, 46]
*Phalaris arundinacea* L.	2.00	0.05	0.11	0.00104	FO,FI	[27–29]
*Phragmites australis* Trin. ex Steud.	3.71	0.13	0.46	0.03693	ED,FI,ME,LI,EN,OT,OR	[27, 29, 45, 46, 62, 64, 65]
*Phragmites karka* (Retz.) Trin. ex Steud.	2.00	0.04	0.07	0.00070	ME,FI,EN	[28, 46]
*Phyla nodiflora* (L.) Greene	1.00	0.05	0.05	0.00026	ME	[28, 29, 46]
*Phytolacca acinosa* Roxb.	2.75	0.07	0.20	0.00510	ED,ME,PE,ID	[27–29, 69]
*Pilea notata* C. H. Wright	1.20	0.09	0.11	0.00174	ME,FO	[27–29, 58, 46]
*Pistia stratiotes* L.	1.50	0.14	0.21	0.00835	ME,FO,GR	[27–29, 46, 58, 57, 59, 62]
*Plantago asiatica* L.	1.20	0.09	0.11	0.00261	ME,FO,ED	[28, 46, 57, 65, 70]
*Plantago lanceolata* L.	1.50	0.04	0.05	0.00035	ME,FO	[28, 46]
*Plantago major* L.	1.00	0.09	0.09	0.00145	ME,ED	[28, 46, 69, 70, 76]
*Pluchea indica* (L.) Less.	1.33	0.05	0.07	0.00070	ED,ME	[28, 29, 46]
*Poa acroleuca* Steud.	1.00	0.02	0.02	0.00003	FO	[28]
*Pogonatherum crinitum* Kunth	1.33	0.05	0.07	0.00070	ME,FO	[28, 29, 46]
*Polygonum amphibium* L.	1.00	0.05	0.05	0.00026	ME	[28, 46, 55]
*Polygonum aviculare* L.	1.50	0.14	0.21	0.01391	ED,ME,FO,ID,PE	[28, 29, 46, 57, 58, 67, 70, 74]
*Polygonum barbatum* L.	1.00	0.04	0.04	0.00012	ME	[28, 46]
*Polygonum capitatum* Buch.-Ham. ex D. Don	1.00	0.07	0.07	0.00046	ME	[28, 29, 46, 58]
*Polygonum chinense* L.	1.00	0.05	0.05	0.00026	ME	[28, 29, 46]
*Polygonum excurrens* Steward	1.00	0.02	0.02	0.00003	ME	[55]
*Polygonum hydropiper* L.	1.41	0.30	0.43	0.07096	ME,ED,FO,OT,PE,ID	[28, 29, 45, 46, 50–53, 55, 57–59, 62, 63, 67, 70, 75]
*Polygonum japonicum* Meisn.	1.17	0.11	0.13	0.00244	ME,PE	[27–29, 46, 55, 58]
*Polygonum jucundum* Meisn.	1.00	0.04	0.04	0.00023	ME,PE	[55, 58]
*Polygonum kawagoeanum* Makino	1.00	0.02	0.02	0.00003	ME	[58]
*Polygonum lapathifolium* L.	2.13	0.14	0.30	0.03154	ED,ME,FO,PE,LI,ID,EN,GR	[27, 46, 50, 55, 57, 58, 62, 67]
*Polygonum lapathifolium* var. *salicifolium* Sibth.	2.50	0.04	0.09	0.00116	ME,PE,LI,ID	[55, 58]
*Polygonum longisetum* var. *rotundatum* A. J. Li	1.25	0.07	0.09	0.00116	ME,ID	[27, 46, 55, 58]
*Polygonum macranthum* Meisn.	1.00	0.04	0.04	0.00012	ME	[55, 58]
*Polygonum nepalense* Meisn.	1.00	0.05	0.05	0.00026	ME	[28, 46, 58]
*Polygonum orientale* L.	2.60	0.18	0.46	0.05276	ED,ME,FO,PE,LI,OR,ID	[27–29, 45, 46, 55, 57–59, 62]
*Polygonum perfoliatum* L.	2.50	0.07	0.18	0.00348	ME,PE,ID	[27, 28, 46, 58]
*Polygonum persicaria* L.	1.33	0.05	0.07	0.00104	ME,FO,PE	[28, 58, 67]
*Polygonum posumbu* Buch.-Ham. ex D. Don	1.00	0.02	0.02	0.00003	ME	[58]
*Polygonum sibiricum* Laxm.	2.00	0.02	0.04	0.00012	ME,FO	[28]
*Polygonum sieboldii* Meisn.	1.00	0.05	0.05	0.00026	ME	[28, 29, 46]
*Polygonum taquetii* H. Lév.	1.00	0.02	0.02	0.00003	ME	[58]
*Polygonum thunbergii* Siebold & Zucc.	2.00	0.05	0.11	0.00209	ED,ME,FO,ID	[28, 46, 58]
*Polygonum viscosum* Buch.-Ham. ex D. Don	1.67	0.05	0.09	0.00130	ED,ME,ID	[28, 58, 63]
*Potamogeton crispus* L.	2.00	0.11	0.21	0.00835	ED,ME,FO,GR	[27, 29, 35, 46, 62, 64]
*Potamogeton cristatus* Regel & Maack	1.00	0.04	0.04	0.00023	ME,FO	[46, 62]
*Potamogeton distinctus* A. Benn.	1.33	0.05	0.07	0.00070	FO,GR	[27, 28, 62]
*Potamogeton lucens* L.	1.00	0.04	0.04	0.00012	GR	[27, 62]
*Potamogeton maackianus* A. Benn.	1.00	0.02	0.02	0.00003	FO	[62]
*Potamogeton malaianus* Miq.	2.00	0.05	0.11	0.00104	FO,GR	[27, 28, 62]
*Potamogeton natans* L.	1.00	0.05	0.05	0.00078	ME,FO,GR	[46, 54, 64]
*Potamogeton octandrus* Poir.	1.00	0.02	0.02	0.00003	FO	[62]
*Potamogeton oxyphyllus* Miq.	1.00	0.02	0.02	0.00003	FO	[62]
*Potamogeton pectinatus* L.	1.60	0.09	0.14	0.00348	ME,FO,GR	[27–29, 46, 62]
*Potamogeton perfoliatus* L.	1.00	0.02	0.02	0.00003	ME	[46]
*Potamogeton pusillus* L.	2.00	0.04	0.07	0.00046	FO,GR	[27, 62]
*Potentilla anserina* L.	2.17	0.11	0.23	0.01357	ED,ME,FO,ID,OT,LI	[28, 29, 46, 70, 69, 79]
*Potentilla discolor* Bunge	1.33	0.05	0.07	0.00070	ED,ME	[28, 29, 46]
*Potentilla flagellaris* D. F. K. Schltdl.	2.00	0.04	0.07	0.00093	ED,ME,FO,GR	[28, 29]
*Potentilla kleiniana* Wight & Arn.	1.00	0.05	0.05	0.00026	ME	[28, 29, 46]
*Potentilla reptans* L.	1.50	0.04	0.05	0.00035	ED,ME	[28, 46]
*Prunella vulgaris* L.	1.00	0.05	0.05	0.00026	ME	[28, 29, 46]
*Pseudoraphis sordida* (Thwaites) S. M. Phillips & S. L. Chen	1.00	0.04	0.04	0.00012	FO	[27, 29]
*Ranunculus cantoniensis* DC.	1.00	0.07	0.07	0.00046	ME	[28, 29, 46, 58]
*Ranunculus chinensis* Bunge	1.17	0.11	0.13	0.00244	ME,PE	[27–29, 46, 58, 68]
*Ranunculus japonicus* Thunb.	1.17	0.11	0.13	0.00244	ME,PE	[27–29, 46, 58, 74]
*Ranunculus sceleratus* L.	1.00	0.09	0.09	0.00072	ME	[28, 29, 46, 58, 59]
*Ranunculus sieboldii* Miq.	1.00	0.07	0.07	0.00046	ME	[27–29, 46]
*Ranunculus ternatus* Thunb.	1.00	0.09	0.09	0.00072	ME	[27–29, 46, 58]
*Reynoutria japonica* Houtt.	2.50	0.04	0.09	0.00116	ED,ME,PE,ID	[28, 46]
*Roegneria ciliaris* (Trin.) Nevski	1.00	0.05	0.05	0.00026	FO	[28, 29, 57]
*Rorippa dubia* (Pers.) Hara	2.50	0.04	0.09	0.00116	ED,ME,FO,ID	[28, 29]
*Rorippa globosa* (Turcz.) Hayek	1.60	0.09	0.14	0.00348	ED,FO,ID	[27, 28, 58, 62, 67]
*Rorippa islandica* (Oeder) Borbás	3.33	0.05	0.18	0.00348	ED,ME,FO,ID	[27, 28, 45]
*Rotala indica* Koehne	1.00	0.07	0.07	0.00139	ED,ME,FO	[27, 28, 63, 62]
*Rotala rotundifolia* (Buch.-Ham. ex Roxb.) Koehne	1.00	0.07	0.07	0.00093	ME,FO	[46, 75, 29, 62]
*Rumex acetosa* L.	3.17	0.11	0.34	0.01652	ED,ME,FO,PE,ID	[27–29, 46, 58, 65]
*Rumex crispus* L.	1.00	0.09	0.09	0.00145	ED,ME	[28, 30, 46, 67, 79]
*Rumex dentatus* L.	1.75	0.07	0.13	0.00244	ME,FO,PE	[27, 28, 46, 62]
*Rumex japonicus* Houtt.	2.60	0.09	0.23	0.00942	ED,ME,FO,ID,LI	[28, 29, 46, 57, 58]
*Rumex maritimus* L.	1.33	0.05	0.07	0.00070	ME,FO	[28, 29, 63]
*Rumex nepalensis* Spreng.	1.25	0.07	0.09	0.00116	ME,ID	[28, 29, 46, 68]
*Rumex patientia* L.	2.50	0.04	0.09	0.00116	ED,ME,ID,LI	[28, 46]
*Rungia chinensis* Benth.	1.00	0.02	0.02	0.00003	ME	[28]
*Saccharum spontaneum* L.	3.00	0.05	0.16	0.00313	FI,FO,OT,EN	[27–29]
*Sacciolepis indica* (L.) Chase	1.00	0.04	0.04	0.00012	FO	[27, 28]
*Sacciolepis myosuroides* (R. Br.) A.Camus	1.00	0.04	0.04	0.00012	FO	[28, 29]
*Sagina japonica* (Sw. ex Steud.) Ohwi	1.00	0.07	0.07	0.00046	ME	[27, 28, 46, 58]
*Sagittaria pygmaea* Miq.	1.25	0.07	0.09	0.00174	ME,FO,GR	[27–29, 46]
*Sagittaria trifolia* L.	1.88	0.14	0.27	0.01739	ED,ME,FO,LI,OR	[27–29, 45, 46, 63, 65, 64]
*Salicornia europaea* L.	2.50	0.04	0.09	0.00087	ME,ID,EN	[27, 28]
*Salvia plebeia* R. Br.	1.00	0.07	0.07	0.00046	ME	[27–29, 46]
*Salvinia natans* (L.) All.	1.89	0.16	0.30	0.01331	ME,FO,GR	[27–30, 46, 57, 58, 62, 64]
*Saururus chinensis* Hort. ex Loudon	1.33	0.05	0.07	0.00070	ME,GR	[28, 29, 46]
*Scirpus juncoides* Roxb.	1.50	0.04	0.05	0.00035	ME,FI	[28, 46]
*Scirpus planiculmis* F.Schmidt	2.75	0.07	0.20	0.00765	ED,ME,FI,FO,LI,EN	[28, 57, 62, 67]
*Scirpus tabernaemontani* Salzm. ex Ball	2.50	0.11	0.27	0.01304	ME,FI,FO,EN,OR	[27, 28, 45, 57, 64, 65]
*Scirpus triangulatus* Roxb.	1.40	0.09	0.13	0.00304	ME,FO,FI	[28, 29, 45, 46, 67]
*Scirpus triqueter* L.	1.43	0.13	0.18	0.00406	FI,FO	[27–29, 45, 57, 62, 64]
*Scirpus wallichii* Nees	1.00	0.02	0.02	0.00003	ME	[28]
*Scirpus yagara* Ohwi	2.57	0.13	0.32	0.01826	ME,FI,FO,ID,LI	[27, 28, 45, 46, 59, 62, 64]
*Scrophularia ningpoensis* Hemsl.	1.00	0.05	0.05	0.00026	ME	[28, 29, 46]
*Sesbania cannabina* (Retz.) Poir.	2.67	0.05	0.14	0.00417	ME,FI,FO,GR,EN,ID	[28, 29, 46]
*Sinosenecio oldhamianus* (Maxim.) B. Nord.	2.00	0.04	0.07	0.00046	FO,GR	[28, 57]
*Sium suave* Walter	1.00	0.07	0.07	0.00093	ME,FO	[28, 29, 46, 64]
*Solanum torvum* Sw.	1.33	0.05	0.07	0.00070	ED,ME	[28, 29, 46]
*Sparganium stoloniferum* (Graebn.) Buch.-Ham. ex Juz.	1.78	0.16	0.29	0.02505	ME,OR,FO,FI,GR,OT	[27–29, 45, 46, 57, 59, 62, 64]
*Spilanthes paniculata* Wall.	1.00	0.05	0.05	0.00026	ME	[28, 29, 46]
*Spirodela polyrhiza* (L.) Schleid.	1.50	0.18	0.27	0.01304	ME,FO,GR	[27–29, 46–48, 59, 63, 64, 62]
*Stachys adulterina* Hemsl.	1.75	0.07	0.13	0.00162	ED,ME	[27, 46, 58, 61]
*Stachys chinensis* Bunge ex Benth.	1.00	0.04	0.04	0.00012	ME	[46, 64]
*Stachys japonica* Miq.	1.75	0.07	0.13	0.00325	ED,ME,ID,LI	[27–29, 58]
*Stachys oblongifolia* Wall.	1.33	0.05	0.07	0.00070	ME,FO	[28, 29, 46]
*Stellaria uliginosa* Murray	1.00	0.04	0.04	0.00023	ME,FO	[28, 46]
*Stephania japonica* (Thunb.) Miers	1.00	0.09	0.09	0.00072	ME	[27–29, 46, 80]
*Suaeda glauca* Bunge	1.60	0.09	0.14	0.00464	ED,ME,ID,OT	[27–29, 46, 70]
*Suaeda salsa* Pall.	2.00	0.05	0.11	0.00209	ED,FO,GR,ID	[27, 29, 70]
*Thalictrum simplex* L.	1.00	0.05	0.05	0.00026	ME	[28, 29, 46]
*Trapa bicornis* L. f.	1.67	0.16	0.27	0.01957	ED,ME,FO,ID,LI	[27, 29, 45, 46, 57, 58, 62, 65, 73]
*Trapa bispinosa* Roxb.	2.33	0.05	0.13	0.00244	ED,ME,FO,LI	[28, 29, 46]
*Trapa incisa* Siebold & Zucc.	1.50	0.07	0.11	0.00278	ED,ME,FO,ID	[27, 29, 46, 65]
*Trapa maximowiczii* Korsh.	1.50	0.04	0.05	0.00035	ED,LI	[28, 29]
*Trifolium pratense* L.	2.50	0.04	0.09	0.00116	ME,FO,GR,ID	[28, 46]
*Typha angustata* Bory & Chaub.	1.50	0.04	0.05	0.00035	ME,FI	[28, 46]
*Typha angustifolia* L.	1.63	0.14	0.23	0.00904	ED,ME,FI	[27–29, 45, 46, 64, 65, 67]
*Typha latifolia* L.	2.17	0.11	0.23	0.00904	FI,ME,ED,OR	[28, 29, 46, 64, 65, 70]
*Typha orientalis* C. Presl	2.13	0.14	0.30	0.01577	ME,FI,ED,OR	[27–29, 33, 34, 46, 59, 64]
*Typhonium giganteum* Engl.	1.00	0.05	0.05	0.00026	ME	[28, 29, 46]
*Urtica angustifolia* Fisch. ex Hornem.	2.33	0.05	0.13	0.00244	ED,ME,FI,ID	[28, 29, 46]
*Utricularia aurea* Lour.	1.00	0.04	0.04	0.00023	FO,OR	[58, 60]
*Utricularia vulgaris* L.	1.00	0.02	0.02	0.00003	FO	[64]
*Valeriana flaccidissima* Maxim.	1.00	0.02	0.02	0.00003	ME	[28]
*Valeriana officinalis* L.	1.00	0.05	0.05	0.00026	ME	[28, 29, 46]
*Vallisneria natans* (Lour.) H. Hara	1.33	0.05	0.07	0.00070	ME,FO	[27, 28, 46]
*Verbena officinalis* L.	1.00	0.09	0.09	0.00072	ME	[28, 29, 46, 68, 78]
*Veronica anagallis-aquatica* L.	1.33	0.05	0.07	0.00070	ED,ME	[28, 29, 46]
*Veronica undulata* Wall.	1.00	0.04	0.04	0.00012	ME	[28, 29]
*Vicia bungei* Ohwi	2.00	0.04	0.07	0.00046	FO,GR	[28, 57]
*Viola grypoceras* A.Gray	1.00	0.05	0.05	0.00026	ME	[27–29]
*Viola inconspicua* Blume	1.00	0.09	0.09	0.00072	ME	[27–29, 46, 58]
*Wolffia arrhiza* (L.) Wimm.	1.50	0.07	0.11	0.00209	ED,FO,GR	[27–29, 48]
*Zantedeschia aethiopica* (L.) Spreng.	1.00	0.04	0.04	0.00023	OR,OT	[27, 29]
*Zizania latifolia* Turcz.	3.33	0.16	0.54	0.06262	ED,ME,FI,FO,OT,ID,GR,OR	[27, 29, 39, 45, 46, 57, 62, 64, 65]

UV: Use value; RFC: Relative Frequency of Citation; CI: Cultural Importance Index; CV: Cultural Value Index.

Relative Frequency of Citation (RFC) varied between 0.02 (55 species, nearly 20% of the total) to 0.3 (Table 4). The top three species based on RFC were *Polygonum hydropiper* (RFC = 0.3), *Oenanthe javanica* (RFC = 0.25) and *Mentha haplocalyx* (RFC = 0.21). The next highest score was for *Acorus calamus* (RFC = 0.20) (Table 4). Many species with high RFC scores were likely to be used over extensive geographical areas, while many of those scoring just 0.02 were likely to be used only very locally.

Cultural Importance scores (CI) ranged between 0.02 (48 species, including *Rungia chinensis* and *A. gramineum*) and 0.54 (*E. ferox* and *Z. latifolia*) (Table 4), while those for Cultural Value (CV) ranged from 0.00003 (the same 48 species as for CI) and 0.07096 (*Polygonum hydropiper*) (Table 4). Species with the lowest CI or CV scores had only one kind of use and were mentioned only in one literature source.

**Figure 2:**
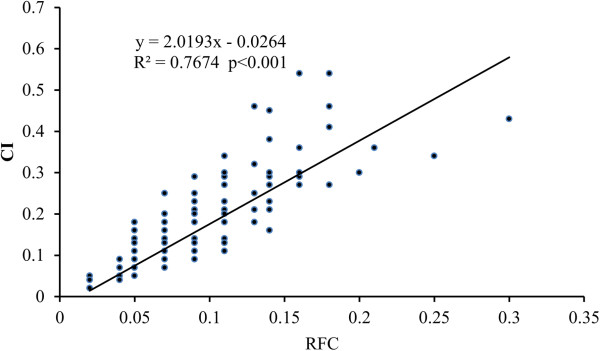
**The correlation between Relative Frequency of Citation** **(RFC)** **and Cultural Importance (CI).**

**Figure 3:**
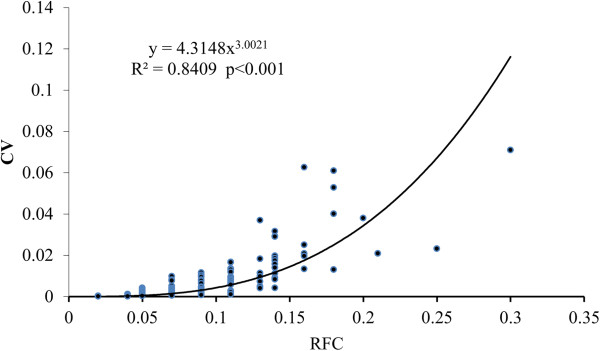
**The correlation between Relative Frequency of Citation** **(RFC)** **and Cultural Value Index (CV).**

**Figure 4:**
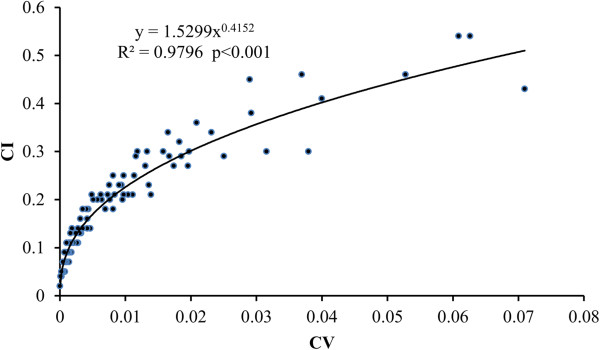
**The correlation between Cultural Value Index** **(CV)** **and Cultural Importance (CI).**

Significant correlations were found between the scores of species for RFC, CI, and CV (Figures 2, 3 and 4), once the data had been normalized appropriately. Five of the top ten species according to RFC also appeared in the top ten lists for CI and CV. These species are *Polygonum hydropiper*, *P. orientale*, *Euryale ferox*, *Zizania latifolia*, and *Coix lacryma*-*jobi*. The 48 species with the lowest CV scores were also lowest according to UV, RFC and CI.

## Discussion

### Diversity of wetland useful plant species

The 350 wetland species recorded as traditionally used in China according to the literature are distributed unevenly across 66 families. The top families are Poaceae, Cyperaceae, Polygonaceae, Lamiaceae, Asteraceae, Rannunculaceae, Hydrocharitaceae and Potamogetonaceae. An uneven distribution of useful wetland species by plant family has also been found elsewhere in the world [11, 88–90], for instance in Manipur (India) where Jain *et al*. found that Polygonaceae, Araceae, Cyperaceae and Poaceae contributed disproportionately to the list of useful species [11]. Coincidentally, many of the top families found in the Manipur study are also dominant or abundant in wetland plant communities in many parts of China [91–96]. Species scoring highly in our study and which also have wide distributions elsewhere in the world, such as *Phragmites australis*, *Polygonum hydropiper* and *Zizania latifolia*, are always mentioned frequently in the literature from other places. All have high UV, CI and CV values according to our study (Table 4). This suggests that families rich in wetland species are more likely to be used than others, the key factor being the local presence of species potentially available for people’s attention and possible use. This result is similar to those reported for other regions [23]. Moerman *et al*. have argued in the case of medicinal plants that the characteristics of the local flora have a big influence on people’s knowledge [97]. The more often people come into contact with particular elements of the flora, the more likely they are to find uses for them. Knowledge about the usefulness of such plants will tend to grow disproportionately, as experience is accumulated. Traditional knowledge is always related to local people’s contact with the local environment [23].

### The characteristics of usage of wetland useful species

Our results show that wetland plants have been used for multiple purposes in most parts of China. The three most important uses are provision of medicine, food and fodder (Table 2), all required regularly by people as they go about their daily lives [11, 13, 98, 99]. Providing people with sources of green manure is a further noteworthy use made of wetland plants, with 53 species being used. Adding fertility to the soil is a basic necessity in China, which remains fundamentally an agricultural country. Providing people with sources of fiber is another regular use made of wetland plants. People in China have had a long history of using plant fiber for making cloth, rope and other articles and a rich store of knowledge about the use of wetland plants for fiber extraction and use has been accumulated by people living in and around wetlands [100].

Besides providing local people with material necessities for their everyday lives, wetland plants also provide other products used less frequently, as well as a range of services. Some plants are used as ornamentals, such as *Polygonum orientale. Phragmites australis*, *Miscanthus sinensis*, *Miscanthus sacchariflorus*, while others are important for the strengthening of embankments and protecting soil erosion. Twenty-two species provide raw materials for making wine. China has a cornucopia of traditional knowledge relating to liquor-making; our results confirm that a substantial part of this knowledge relates to wetland species, even though much of this knowledge is historical and not known by current generations. Wetlands can be breeding grounds for mosquitoes and other nuisance insects, reducing agricultural production or transmitting disease, so considerable traditional knowledge of wetland plants relating to pesticides may yet prove to be useful in the modern world. There are also some species having important cultural values, for example the flowers of *Zantedeschia aethiopica* used commonly in sacrificial rites.

Compared with the uses mentioned above, the use of wetland plants for industrial purpose is comparatively recent. Industrially, wetland plants are mostly used as sources of industrial raw materials. For instance, *Scirpus yagara* is used as a raw material in the production of ethyl alcohol and glycerol, while *Mentha haplocalyx* can be a source of volatile oils. These plants can be important source of cash for local people.

Wetland plants provide people with many types of products valuable for subsistence living. The wealth of traditional knowledge that has accumulated about the uses of wetland plants is a reflection of the close relationships traditionally existing between people and their local environments, in this case specifically relating to wetlands. Much of this knowledge is disappearing today along with the loss of traditional lifestyles and retreat of wetlands. Systematic ethnobotanical surveys of traditional knowledge relating to wetlands are therefore needed, while such knowledge still exists.

### Comparison of some quantitative indexes

An increasing number of papers have appeared over recent years discussing the use of quantitative methods in ethnobotanical research [101, 102]. In particular, many new parameters have been suggested for evaluating the cultural importance or significance of plants and determine information consensus between informants [24–26, 103]. The use of such indexes can not only advance the development of ethnobotany, but can also make it possible to compare results between different regions or cultural groups, as well as undertaking meta-analyses.

Use Value (UV) is one of the most frequently used indexes for evaluating ‘the relative usefulness of plants to people’ [23, 24, 83, 84]. It has been successfully applied in many contexts [104–108]. With respect to an analysis of the literature, such as that here, UV reflects not only the number of uses made of a plant as well as the number of literature sources mentioning it. So a plant with high UV value does not necessarily mean that it has multiple uses nor that it is necessarily mentioned in many publications, as we have discussed in an earlier paper [109]. To illustrate this point, three species (*Najas graminea*, *Potamogeton pusillus* and *Monochoria korsakowii*) were all found to have UV = 2 in the present study, but actually the first two of these are only mentioned in two literature sources with two uses in each case, while the third is mentioned in 8 sources but only for one type of use. Among those plants with UV = 1, they have the same total numbers of different uses recorded in the literature and the numbers of literature recording these uses. Although their UV values are the lowest, it does not mean that they have few uses. However, some plants with higher UV values are indeed versatile, such as *P. australis*, *Z. latifolia*, and *N. tetragona*. These plants have a common feature: mentioned by a higher number of literature. So the UV value in a literature study may give us a bias. When using UV index to evaluate a plant, we should use the number of the literature recorded it for reference.

According the formula used for calculating FUV, we can find that FUV depends on the UV of species in a family. So FUV has a similar shortcoming to UV.

Compared with UV, RFC, CI and CV have considered more factors that may lead to a bias. RFC is as same as %P designed in one of our previous papers [109]. Although it has considered the number of the literature which mentions a given species and the total number of literature concerned in the study, it does not take into account the number of uses mentioned in the literature. It just reflects the frequency of a species mentioned by the literature. There are significantly positive correlations between RFC and CI (R^2^ = 0.767, p < 0.001) and CV (R^2^ = 0.841, p < 0.001), respectively (Figure 2 and 3). Because RFC does not consider the number of uses, it will not show the difference of the importance and use values between species. Compared with RFC, CI and CV are two more comprehensive indexes. They consider not only the frequency cited by the literature, but also the number of uses recorded in the literature. There is a significant correlation between CI and CV (R^2^ = 0.980, p < 0.001). The species with higher CI values often have higher CV, such as *P. hydropiper* and *Z. latifolia*. Compared with CI, CV is more sensitive to the information recorded in the literature and is more effective to show the differences of use value and frequency being cited in the literature between species.

In fact, many indexes used in quantitative ethnobotany are related to the use categories. The method of use type classification will affect greatly the calculation results. However, it is often very difficult to produce a perfect scheme of use categories which could be closer to the truth. What can we do is to try our best to make the use categories more reasonable.

## Conclusion

The wide distribution of many species of wetland plants [110, 111] makes it possible to gain a general picture of the uses made of such plants on a macro-scale. A principal conclusion from the present study is that the biggest uses of wetland species, in terms of the number of citations in the literature, are for medicine, food and fodder. We conclude that it is whether or not particular species are growing locally that is a major determinant over whether people actually use them. Cultural Value (CV) and Cultural Importance (CI) are judged to be the most useful quantitative indices for providing measures of the relative importance and usefulness of wetland species, based on analyses of citations in literature that is not specifically ethnobotanical. However, such publications cannot provide detailed information about relationships between wetland plants and people, such as details of the ways in which people use and manage them. China is rich in both wetlands and traditional knowledge of wetland plants, but both wetlands and traditional knowledge are rapidly being lost. Traditional knowledge about wetland plants has much to offer for modern needs, such as the sustainable use of wetland plants, conservation and industrial development. We therefore conclude that there is a great need for detailed systematic ethnobotanical studies on wetland plants to be undertaken as a matter of urgency.
